# Epidemiological and Clinical Pattern of Pediatric Supracondylar Fracture of Humerus in A Provincial Hospital: A Descriptive Cross-sectional Study

**DOI:** 10.31729/jnma.6047

**Published:** 2021-01-31

**Authors:** Pramod Joshi, Pravakar Dawadi, Krishna Rana, Navindra Raj Bista, Rishi Bisht, Prakash Kayastha

**Affiliations:** 1 Department of Orthopedics, Seti Provincial Hospital, Dhangadhi, Nepal; 2 Nepalese Army Institute of Health Sciences, Sanobharyang, Kathmandu, Nepal; 3 All Nepal Hospital Private Limited, Kathmandu, Nepal; 4 Department of Anesthesiology, Tribhuvan University Teaching Hospital, Maharajgunj, Kathmandu, Nepal; 5 Department of Orthopedics, National Academy of Medical Sciences, Bir Hospital, Kathmandu, Nepal; 6 Department of Radiology and Imaging, Tribhuvan University Teaching Hospital, Institute of Medicine, Kathmandu, Nepal

**Keywords:** *orthopedics*, *pediatric*, *supracondylar fracture*

## Abstract

**Introduction:**

Supracondylar fractures of humerus are the most common elbow fractures in children consisting of about 15% of all pediatric fractures and more than half of all elbow fractures. A high incidence of nerve injures, and vascular injuries make this fracture a serious injury. Our study aims to study on the clinical and demographic pattern of pediatric supracondylar fracture cases presenting in the hospital retrospectively.

**Methods:**

We conducted a descriptive cross-sectional study in Seti Provincial Hospital in the month of December. The data from the medical record section was retrospectively collected. A whole sampling technique was used. The descriptive statistical analysis was done.

**Results:**

Seven hundred cases were studied, among which the most common age group was found to be 5-10 410 (58.57%). Most of the cases presented in the emergency department 513 (73.28%), and the most common time of presentation was from 3 AM to 6 AM 170 (24.28%).

**Conclusions:**

Supracondylar fracture cases presented as a common injury among pediatric population. It was presented as an emergency more than general cases.

## INTRODUCTION

Approximately ten percent of all pediatric orthopedic injuries are fractures around the elbow joint, with supracondylar fracture of humerus being the second most common fracture in children accounting for about 75% of all injuries around the elbow.^1,2^ The most common age group in which the supracondylar fractures occur is 5-6 years.^3^

A number of complications associated with supracondylar fractures such as immediate neurovascular injury, cubitus varus deformity, compartment syndrome, Volkmann ischemic contracture, and trochlear osteonecrosis make this fracture a serious injury.^4^ Moreover, a study done by Erika et al. reported that the more time is elapsed between injury and medical management, the more hospitalization days are required. Proper initial management prevents such complications.^5^

Our study aims to study the clinical and demographic pattern of pediatric supracondylar fracture cases presenting in the hospital retrospectively.

## METHODS

A descriptive cross-sectional study was conducted in Seti Provincial Hospital in December, which included the record of pediatric supracondylar fracture from the last one year. Ethical approval was taken from the Ethical Review Board of Nepal Health Research Council (Ref 1527).

All the cases of pediatric supracondylar fracture from the last one year meeting inclusion criteria will be included in the study. Since the whole sampling is used, there is no need for sample size calculation. Children age ≤14 years managed surgically with CRPP or ORIF for supracondylar fracture with complete records were included for the study. However, patients age >15 years, incomplete medical records, and supracondylar fracture managed conservatively in the plaster slab were excluded from the study.

A specifically designed structured record review form will be filled by going through the records of admitted cases of pediatric supracondylar fracture. In order to maintain confidentiality and anonymity, the personal identification details were eliminated, and a consecutive reference number to each case was assigned.

The collected data were kept in Microsoft Excel, and the descriptive statistical analysis was done. Frequency and proportion were calculated for the binary data.

## RESULTS

During this study, we studied around 700 cases of pediatric supracondylar fracture presenting over a 1-year duration. Among them, the majority of patients were male, 397 (56.7%). Category 5 caste (Brahmin, Chhetri) constituted the maximum patient distribution 390 (55.71%).

**Figure 1:**
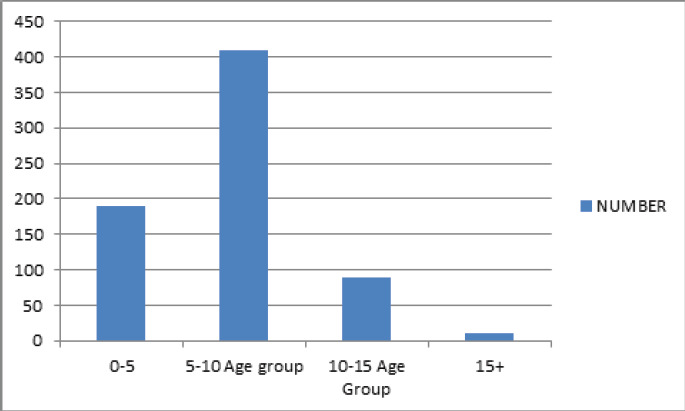
Age group of Supracondylar Fractures Presenting in the hospital.

Among the age group, five to ten age group cases, 410 (58.57%) were the most followed by 0-5 age group 190 (27.14%) (Figure 1).

**Figure 2:**
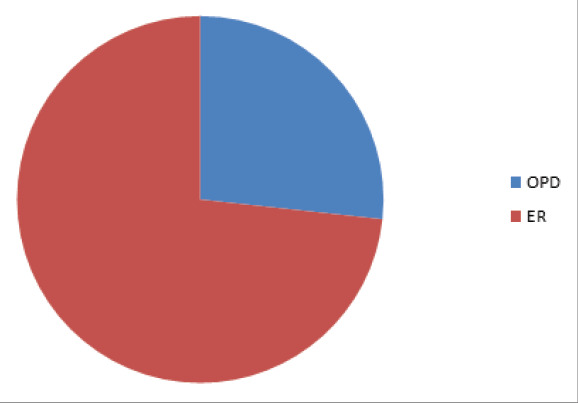
Mode of Presentation of Supracondylar Fracture.

Similarly, most of the cases presented in the emergency department 513 (73.28%), and the most common time of presentation was from 3 AM to 6 AM 170 (24.28%) (Table 1 and Figure 2).

**Table 1:** Time of Presentation of Supracondylar Fracture Cases.

Time of Admission	Number
12 AM - 3 AM	130
3 AM - 6 AM	170
6 AM - 9 AM	90
9 AM - 12 PM	60
12 PM - 3 PM	80
3 PM - 6 PM	20
6 PM - 9 PM	110
9 PM - 12 AM	40

Except for two cases, all the cases 698 (99.71%) presented in their first visit to the hospital. Six hundred and ninety-seven (99.57%) cases were discharged successfully after treatment, and three cases (0.43%) were referred to another center.

## DISCUSSION

In our study, the records of pediatric supracondylar fracture cases over a year have been studied. It showed the common clinical pattern of presentation of cases.

The study done by Barron-Torres et al. showed males to be more commonly affected and highlighted the speed of medical treatment as an important issue. Similarly, our study also showed males to be more commonly affected than females.^5^

Biradar et al. studied type III supracondylar humerus fractures in children treated by closed reduction with percutaneous crossed pin fixation and found the mean age of overall cases to be 5.96 years and the common age group as 4-8 years. In our study, the common age group was found to be 5-10 years.^6^

A retrospective review study done among children with supracondylar humerus fracture by Dhoju et al. showed a mean age of 8.91 years with a range of 2-14 years.^7^ A single center study done by Auso-Perez et al. showed that the presentation risk at nighttime was higher during the summer months.^8^ Our study showed early morning hours (3 AM-6 AM) with a higher load of cases.

This is a single-center retrospective study. Our study may not be truly representative of the epidemiology of orthopedic problems prevalent in the community. As the data are obtained from the records of the ward, if there was an error in the maintained information, it could have caused the loss of several cases. The cross-sectional nature of the study does not allow the establishment of causality.

## CONCLUSIONS

Supracondylar fracture cases presented as a common injury among the pediatric population. It was presented as an emergency for more than general cases. More studies with the inclusion of more sample sizes and more tertiary center are needed for better scientific validity of the study.
